# Frailty dimensions and cognitive function in relation to dual-task cost: A multidomain analysis in community-dwelling older adults

**DOI:** 10.1016/j.tjfa.2026.100166

**Published:** 2026-06-06

**Authors:** Louise Heyzer, Kristabella Low, Cai Ning Tan, Audrey Yeo, Jia Qian Chia, Justin Chew, Joanne Kua, Wee Shiong Lim

**Affiliations:** a Department of Geriatric Medicine, Woodlands Hospital, Singapore; b Institute of Geriatrics and Active Ageing, Tan Tock Seng Hospital, Singapore; c Department of Geriatric Medicine, Tan Tock Seng Hospital, Singapore

**Keywords:** Frailty, Dual-task cost, Executive function, Older adults

## Abstract

**Background:**

Dual-task cost (DTC) is the performance decline observed during simultaneous motor and cognitive tasks. It is associated with cognitive and physiological vulnerability. However, it remains unclear how specific frailty dimensions and cognitive domains contribute to DTC during arithmetic and verbal fluency tasks.

**Objectives:**

We aim to investigate the independent associations between physical, social and oral frailty dimensions versus cognition function with DTC in community-dwelling older adults.

**Methods:**

We studied 281 participants from the GeriLABS-2 study. Physical frailty was assessed using Fried’s Frailty Phenotype, social frailty using the 8-item Social Frailty Scale (SF8) and oral frailty using the Oral Frailty Index-8 (OFI-8). Cognitive function was assessed with Chinese Mini Mental State Examination (CMMSE) and Chinese Frontal Assessment Battery (CFAB). DTC on gait was assessed with arithmetic (counting backwards from 100) and verbal fluency (animal naming) cognitive tasks. Association of DTC with frailty dimensions and cognition were evaluated via hierarchical linear regression, adjusted for demographics.

**Results:**

Higher CFAB score was significantly associated with lower DTC in both arithmetic (B=-2.067, p < 0.001) and verbal fluency (B=-1.489, p = 0.020) tasks, suggesting individuals with better executive function showed lower DTC. Social frailty (Factor 2 – social activity and financial resources) and oral frailty (Factor 3 – dietary and social habits) were significantly associated with DTC for arithmetic task (B = 2.664, p = 0.030; B = 3.718, p = 0.007). No significant association was observed for physical frailty. The final composite model showed that CFAB has a significant associated with DTC arithmetic (B=-2.012, p < 0.001) and DTC verbal fluency (B=-1.537, p = 0.016) tasks.

**Conclusion:**

Cognitive frailty, particularly executive dysfunction, is a key factor contributing to difficulties with dual tasking. Social and oral frailty factors may influence DTC during arithmetic tasks, however their effects were attenuated after adjusting for cognition. Interventions addressing cognitive frailty may be a promising target for mitigating DTC in older adults.

## 1 Introduction

Dual tasking is that ability to perform two tasks simultaneously. It is important for daily functioning in the elderly and impairments in dual-tasking may lead to an increased risk of falls, reduced functional mobility, and declines in cognitive and motor performance. Dual task cost (DTC) refers to the performance decline observed during simultaneous motor and cognitive tasks. It is expressed as a percentage change in the parameters of interest of dual-task versus single-task [1]. Positive values on DTC, which are more commonly seen, often reflect a performance decrement – the individual performing worse on the task when multitasking than when performing it alone [2]. Higher DTC values may indicate increased fall risk or increased cognitive vulnerability. With our aging population increasing, understanding mechanisms underlying dual-task impairment becomes important to reduce falls risk and preserve functional dependence in older adults.

The concept of cognitive frailty (CF) is also relevant to this study. CF is defined as the coexistence of physical frailty and mild cognitive impairment in the absence of dementia [3], and has been reported in approximately 1–22% of community-dwelling older adults depending on the criteria used [4]. Previous research has established the role of executive function and other cognitive domains including memory, processing speed, and visuospatial ability in relation to physical frailty and multidimensional frailty phenotypes [5]. Individuals with CF are more vulnerable to tasks requiring simultaneous cognitive and motor processing. Thus, Dual-task paradigms may provide a means of detecting early cognitive-motor decline and identifying individuals at a stage where intervention remains possible.

Physical frailty [6] and cognition [7] are traditionally associated with the dual-tasking ability, however gaps remain in our understanding of the relationship with the other frailty dimensions. Frailty is a state of increased vulnerability to adverse health outcomes when exposed to a stressor [8]. In the Asia-Pacific region, the prevalence of frailty in community-dwelling older adults is 3.5%−27% [9]. While physical frailty has been widely studied in terms of gait and movement decline, less is known how social and oral frailty interact and impact on the cognitive-motor performance in cognitively demanding situations like dual-tasking.

Among the frailty dimensions, social frailty is relatively unexplored. Bunt’s concept of Social Production Function theory defined it as a continuum of being at risk of losing, or having lost, resources that are important for fulfilling one or more basic social needs during the life span [10]. A previous study [11] highlighted the association of social frailty with executive dysfunction, particularly the cognitive domain, suggesting the importance of better understanding the complex interactions between social frailty and cognitive-motor performance as a possible target for further intervention.

Oral frailty is defined as the age-related functional decline of orofacial structures [12]. It encompasses swallowing difficulties, dental function, and dietary habits. The Japan Dental Association has described oral frailty as a risk factor for poor nutrition and sarcopenia [13], ultimately leading to a decrease in physical and mental function.

Despite emerging associations, few studies have concurrently assessed all three frailty domains alongside cognitive and dual-task measures [14]. This study addresses this gap using a multidomain approach in a cohort of community-dwelling older adults. The primary aim was to examine the independent associations of physical, social and oral frailty dimensions, and cognitive function with DTC during arithmetic and verbal fluency tasks. We hypothesised that executive dysfunction would be the dominant predictor of DTC across both tasks, given its established role in attentional resource allocation. Better understanding of this complex relationship may reveal modifiable risk factors and support multidimensional assessments in aging populations.

## 2 Methods

### 2.1 Study population

**Fig. 1:**
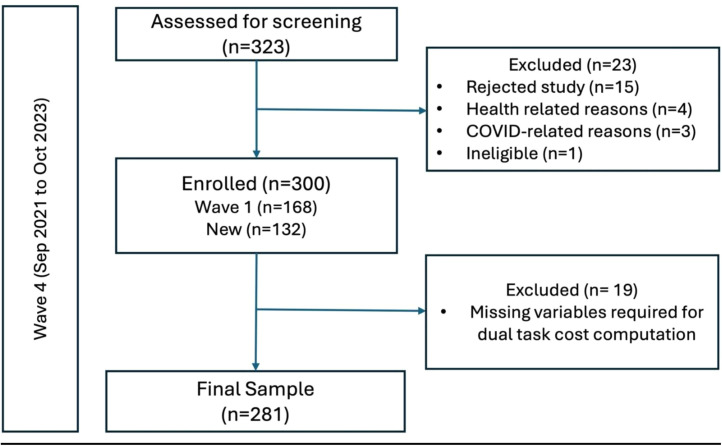
STROBE flow diagram showing participant inclusion for dual task cost analysis.

The “Longitudinal Assessment of Biomarkers for characterization of early Sarcopenia and Osteosarcopenic Obesity in predicting frailty and functional decline in community-dwelling Asian older adults Study” (GeriLABS-2) is a prospective cohort study. This analysis is a cross-sectional study of the baseline data of GeriLABS-2. It involves cognitively intact and functionally independent community-dwelling adults aged 50 and older. The study design has been previously described [15,16]. The exclusion criteria were cognitive impairment (prior diagnosis of dementia or scored ≤ 21 on the modified Chinese Mini-Mental State Examination (CMMSE) [17], inability to walk 8 m independently or resident of a long-term care institution. From this study, we performed cross-sectional analysis of 323 participants recruited from wave 4 visit between September 2021 to October 2023 (Fig. 1). From 323 participants who were screened, 23 participants were excluded to give a final sample of 300. From the 300 enrolled participants, 19 participants were excluded due to missing data on one or more key vriables required for dual task cost computation. This resulted in a final analytic sample of 281 participants. Ethical approval was obtained from the domain-specific review board of the National Healthcare Group (NHG DSRB Reference: 2017/00850). All participants gave written consent prior to participating in the study.

### 2.2 Fried’s frailty phenotype

Physical frailty was assessed with Fried’s Frailty Phenotype, a validated tool that captures early physiological vulnerability [18]. The five components are (1) unintentional weight loss (BMI <18.5kg/m² or self-reported weight loss of >4.5 kg in the past year), (2) fatigue (assessed using two items from the Center for Epidemiologic Studies Depression (CES-D) scale, with positive responses (≥moderate) on either ‘I felt that everything I did was an effort’ or ‘I could not get going’ indicating criterion fulfilment), (3) low physical activity (determined using the Frenchay Activities Index (FAI), with participants in the lowest quintile classified as having low activity), (4) slowness (assessed using usual gait speed over a 6-metre walk, with a cut-off of <1.0 m/s) and (5) weakness (assessed using handgrip strength measured with a Jamar dynamometer, applying Asian Working Group for Sarcopenia cut-offs (men <28 kg, women <18 kg)). Participants were categorised as robust (0 components), pre-frail (1–2 components), or frail (≥3 components).

### 2.3 8-item Social Frailty Scale (SFS-8)

Social frailty was assessed with the locally validated eight-item Social Frailty Scale (SFS-8) that is guided by Bunt’s framework [7,16]. It has a total score of 0–8 points [19]. A score of 0–1 indicates social non-frailty (SNF), 2–3 indicates social pre-frailty (SPF) and 4 or more indicates social frailty (SF). It comprises of three domains – social resources, social activity and financial resources, and social need fulfilment [19].

### 2.4 Oral Frailty Index -8 (OFI-8)

Oral frailty was assessed with the eight-item Oral Frailty Index-8 (OFI-8) with total score of 0–8 points [20]. A score of 0–1 indicates low risk, 2–3 indicates moderate risk and 4 or more indicates high risk of oral frailty. It comprises of three domains – swallowing difficulties, dental care, and dietary and social habits.

### 2.5 Cognitive assessments

Cognitive function was assessed with the CMMSE and Chinese Frontal Assessment Battery (CFAB) [21]. The CMMSE is a 28-question assessment that covers six specific domains of cognitive function: orientation to time, orientation to place, registration, attention and calculation, recall and language and praxis [22]. The locally validated CFAB is a six-item assessment of executive function that comprises two domains (cognitive and behavioural control). The cognitive control assesses conceptualisation, mental flexibility and motor programming. The behavioural control assesses sensitivity to interference, mental flexibility and environmental autonomy [23].

### 2.6 Covariates

Baseline data included age, gender, education, assessed body mass index (BMI) and comorbidities. Co-morbities included diabetes mellitus, hypertension, hyperlipidaemia and number of vascular risk factors. Functional ability was assessed by Barthel’s index for basic activities of daily living (ADL) [24] and Lawton and Brody’s index for instrumental ADL [25].

### 2.7 Dual-tasking cost and outcomes measures

Dual Task Cost (DTC) was assessed using two dual-tasking conditions, each combining a cognitive task while walking. The cognitive tasks differed in their primary demands: (1) arithmetic (serial subtraction), in which participants counted backwards from 100 to 1 while walking, engaging working memory, attention, and executive control; and (2) verbal fluency (animal naming), in which participants named as many animals as possible while walking, primarily engaging semantic retrieval and language processes.

The gait task involved walking 6 m along a corridor with a 2 m acceleration phase. Each condition was performed once under single-task (walking only) and dual-task (walking with cognitive task) conditions, with order counterbalanced. Gait speed (m/s) was recorded using a stopwatch, with timing taken over the central walkway to minimise acceleration and deceleration effects.

Single-task gait speed was assessed by asking participants to walk the same course at their usual pace without a concurrent cognitive task. This served as the reference measure of baseline gait performance for comparison with dual-task conditions.

DTC was calculated as: DTC (%) = [(Single-task gait speed – Dual-task gait speed) / Single-task gait speed] × 100. Higher DTC indicates greater decline in performance under multitasking conditions.

DTC from the verbal fluency (DTC-VF) and arithmetic (DTC-A) tasks were analysed.

### 2.8 Statistical analysis

We performed statistical analyses using IBM SPSS version 31.0 (IMB Corporation, Armonk, NY, USA). All statistical tests were two-tailed, with p < 0.05 considered statistically significant.

Descriptive statistics were presented as means ± standard deviations (SD), medians (IQR), or frequencies (%), as appropriate. Between-group differences (normal vs high DTC) were compared using t-tests, Mann-Whitney U, or chi-square tests.

For descriptive and univariate comparisons, participants were classified into ‘high DTC’ (top quintile, ≥80th percentile) and ‘normal DTC’ groups. The 80th-percentile threshold was selected a priori to identify individuals with the greatest degree of cognitive-motor interference while maintaining adequate group sizes for comparison (high DTC n = 56). Quintile-based categorisation is widely used in epidemiological research to define high-risk subgroups in the absence of established clinical cutoff [26]. All hierarchical regression analyses treated DTC as a continuous outcome variable.

We conducted hierarchical linear regression in three models. Model 1 adjusted for cognitive predictors (Table 2), model 2 adjusted for frailty predictors (Table 3), whilst the final model (Model 3) adjusted for frailty and cognitive predictors that were significant from earlier models(Table 4). All models were adjusted for age, gender and years of education.

## 3 Results

A total of 281 participants were included in this analysis (Fig. 1). They were categorized into normal and high DTC groups for both verbal fluency and arithmetic tasks. Those in the top quintile of DTC distribution for each task were defined as “high DTC group” while all others comprised the “normal DTC group”. This approach was chosen to capture individuals most vulnerable to dual-task impairment. The top quintile threshold was selected to allow clearer contrasts in univariate analyses and ensuring sufficient group sizes for statistical comparison. Baseline characteristics are summarized in Table 1.

**Table 1:** Baseline characteristics *p < 0.05.

	DTC animal			DTC subtraction		
Characteristic	Normal (n = 225)	High (n = 56)	p-values	Normal (n = 225)	High (n = 56)	p-values
**Demographics**						
Age (years)	67.21±7.18	66.75±7.02	0.717	66.96±7.18	67.82±6.97	0.419
Gender (Female)	158 (70.2%)	37 (66.1%)	0.546	154 (68.4%)	41 (73.2%)	0.488
Years of education	11.92±3.79	11.94±4.15	0.982	12.18±3.85	10.93±3.74	0.030*
BMI (kg/m²)	23.32±3.07	23.25±2.85	0.871	23.33±3.10	23.20±2.75	0.768
**Health-Related Covariates**						
Diabetes	35 (15.6%)	6 (10.7%)	0.358	36 (16.0%)	5 (8.9%)	0.180
Hypertension	71 (31.6%)	18 (32.1%)	0.933	67 (29.8%)	22 (39.3%)	0.171
Hyperlipidaemia	112 (49.8%)	23 (41.1%)	0.243	109 (48.4%)	26 (46.4%)	0.787
Vascular risk factors (0–8)	4 (3–5)	4 (3–4.75)	0.481	4 (3–5)	4 (3–5)	0.497
**Frailty Dimensions**						
**Physical Frailty (Fried Criteria)**						
Not Frail	86 (38.2%)	25 (44.6%)	0.384	91 (40.4%)	20 (35.7%)	0.784
Pre-Frail	120 (53.3%)	29 (51.8%)		117 (52.0%)	32 (57.1%)	
Frail	19 (8.4%)	2 (3.6%)		17 (7.6%)	4 (7.1%)	
Fried Frailty Score (0–5)	1 (0–1)	1 (0–1)	0.250	1 (0–1)	1 (0–2)	0.443
**Social Frailty (SF-8 Total)**						
Not Frail	150 (66.7%)	36 (64.3%)	0.042	153 (68.0%)	33 (58.9%)	0.044*
Pre-Frail	66 (29.3%)	13 (23.2%)		63 (28.0%)	16 (28.6%)	
Frail	9 (4.0%)	7 (12.5%)		9 (4.0%)	7 (12.5%)	
SF8 Total Score	1 (0–2)	1 (0–2)	0.179	1 (0–2)	1 (0.25 −2.75)	0.025*
**Oral Frailty (OFI-8)**						
Low	144 (64.0%)	35 (62.5%)	0.850	144 (64.0%)	35 (62.5%)	0.303
Moderate	36 (16.0%)	8 (14.3%)		38 (16.9%)	6 (10.7%)	
High	45 (20.0%)	13 (23.2%)		43 (19.1%)	15 (26.8%)	
OFI-8 Total Score	1 (0–3)	1.5 (0–3)	0.729	1 (0–3)	2 (1–4)	0.389
Outcome characteristics						

	DTC animal			DTC subtraction		
Characteristic	Normal (n = 225)	High (n = 56)	p-values	Normal (n = 225)	High (n = 56)	p-values
**Functional Status**						
Total ADL	100 (100–100)	100 (95–100)	0.028*	100 (100–100)	100 (95–100)	0.018*
Total iADL	23 (22–23)	23 (22–23)	0.847	23 (22–23)	23 (22–23)	0.623
**Physical performance**						
SPPB (0–12)	12 (11–12)	12 (11–12)	0.433	12 (11–12)	12 (11–12)	0.099
Gait speed (m/s)	1.13±0.211	1.24±0.28	0.003*	1.15±0.22	1.16±0.27	0.713
Grip strength (kg)	26.90±7.68	27.39±7.96	0.669	27.38±7.72	25.43±7.61	0.091
**Cognition**						
CMMSE						
CMMSE Total Score	26 (25–27)	26 (25–27)	0.507	26 (25–28)	26 (25–27)	0.003*
CMMSE Factor – orientation to time	4 (4–4)	4 (4–4)	0.370	4 (4–4)	4 (4–4)	0.160
CMMSE Factor – orientation to place	4 (4–4)	4 (4–4)	0.815	4 (4–4)	4 (4–4)	0.764
CMMSE Factor – registration	3 (3–3)	3 (3–3)	0.316	3 (3–3)	3 (3–3)	0.316
CMMSE Factor – attention and calculation	5 (4–5)	4 (4–5)	0.648	5 (4–5)	4 (3.25–5)	0.149
CMMSE Factor – recall	3 (2–3)	3 (2–3)	0.984	3 (2–3)	3 (2–3)	0.048*
CMMSE Factor – language and praxis	9 (8–9)	9 (8–9)	0.526	9 (8–9)	9 (8–9)	0.005*
CFAB						
CFAB Total Score	17 (16–18)	17 (16–18)	0.267	17 (16–18)	16 (15–17)	<0.001*
Cognitive Control (Q1+2 + 5)	8 (7–9)	8 (7–9)	0.413	8 (8–9)	7 (6–9)	<0.001*
Behavioral control (Q3+4 + 6)	9 (9–9)	9 (8.25–9)	0.284	9 (9–9)	9 (8–9)	0.003*
**Dual-Task Performance**						
DTC – Verbal Fluency (Animal Naming)	11.53±10.07	36.85±9.73	<0.001*	13.14±12.6	30.37±12.04	<0.001*
DTC – Subtraction Task (100–1)	9.99±10.92	27.91±12.86	<0.001*	8.45±8.86	33.88±8.23	<0.001*

*p < 0.05.

Values are expressed in mean±SD (continuous variables), N(%) (categorical variables) and median (IQR) (non-parametric continuous variables).

There were no significant differences between high and normal DTC groups in age, gender, or BMI for either task. The majority of participants were female (n = 195, 69.4%), with similar proportions across groups for both DTC-VF (70.2%vs 66.1%, p = 0.546) and DTC-A (68.4%vs 73.2%, p = 0.488). Both DTC groups were similar in terms of comorbidities: diabetes, hypertension, hyperlipidaemia and there were no significant differences found in the number of vascular risk factors. The high DTC arithmetic group had significantly fewer years of education (mean 10.93 ± 3.74 years) compared to the normal group (mean 12.18 ± 3.85 years, p = 0.030).

In terms of frailty dimensions, the distribution of physical frailty or oral frailty is not significant between normal and high DTC groups. Social frailty categories (p = 0.044) and total score (p = 0.025) were higher in the high DTC arithmetic tasks group.

Total bADL score was significantly lower in high DTC verbal fluency group (p = 0.028) and high DTC arithmetic group (p = 0.018). Gait speed was unexpectedly faster in high DTC group for verbal fluency (1.24±0.28) compared to the normal group (1.13±0.21, p = 0.003).

For the high DTC arithmetic group, CMMSE (p = 0.003) and CFAB (p < 0.001) were both significantly lower. As expected, DTC was significantly higher in the high DTC groups for both tasks.

### 3.1 Relationship between cognitive measures and dual task cost in verbal fluency and arithmetic tasks

The CMMSE total score was not significantly associated with dual task cost (DTC) in both verbal fluency (B-0.523, p = 0.334) and arithmetic (B-0.785, p = 0.118). However, the CMMSE recall subdomain showed a significant association with DTC arithmetic tasks (B=−2.785, p = 0.022) but not with DTC verbal fluency tasks (B=−1.211, p = 0.353).

The CFAB total score was significantly associated with DTC in both tasks (verbal fluency B=−1.314), p = 0.020; arithmetic (B=−2.067, p < 0.001). CFAB cognitive control showed a trend toward association with DTC verbal fluency tasks (B-1.569, p = 0.055) and was significantly associated with DTC arithmetic tasks (B=−1.599, p = 0.032). CFAB behavioural control was not associated with DTC verbal fluency (B=−1.314, p = 0.300) however was significantly associaed with DTC arithmetic tasks (B=−3.082, p = 0.008). (Table 2)

**Table 2:** CMMSE and CFAB (total and factor scores): associations between cognitive measures and dual task cost in animal naming and subtraction tasks.

	DTC Animal					DTC Subtraction				
	B (Unstd.)	SE	β (Std.)	p-value	R²	B (Unstd.)	SE	β (Std.)	p-value	R²
CMMSE										
CMMSE Total	−0.527	.541	−0.063	0.331	0.004	−0.784	0.501	−0.099	0.119	0.037
CMMSE Factor – orientation to time	−2.710	2.201	−0.075	.0219	0.017	−2.058	2.036	−0.061	0.313	0.052
CMMSE Factor – orientation to place	0.951	2.027	0.030	0.639		−0.196	1.875	−0.006	0.917	
CMMSE Factor – registration	3.990	5.571	0.044	0.474		0.355	5.154	0.004	0.945	
CMMSE Factor – attention and calculation	−0.945	0.939	−0.065	0.315		−0.187	0.869	−0.014	0.830	
CMMSE Factor – recall	−1.194	1.311	−0.057	0.363		−2.831	1.213	−0.143	0.020*	
CMMSE Factor – language and praxis	0.715	1.319	0.035	0.588		0.228	1.220	0.012	0.852	
CFAB										
CFAB Total	−1.489	0.639	−0.158	0.020*	0.021	−2.066	0.585	−0.233	<0.001*	0.072
CFAB Cognitive control	−1.574	0.815	−0.130	0.055	0.021	−1.593	0.745	−0.140	0.033*	0.075
CFAB Behavioral Control	−1.305	1.268	−0.065	0.304		−3.091	1.159	−0.163	0.008*	

*p < 0.05.

### 3.2 Relationship between frailty measures and dual task cost in verbal fluency and arithmetic tasks

Social Frailty - Factor 2 (Social activity and financial resources) was significantly associated with DTC arithmetic tasks (B = 2.664, p = 0.030). The three items that make up Factor 2 are going out less frequently compared with the prior year, eating with someone at least one time a day and limitation by financial resources to pay for needed medical services.

Oral Frailty – Factor 3 (Dietary and Social Habits) was also significantly associated with DTC arithmetic tasks (B = 3.718, p = 0.007). The two items that make up Factor 3 are going out less frequently compared to the last year and feasibility to chew hard food.

No physical frailty component showed significance for DTC arithmetic or verbal fluency tasks. (Table 3)

**Table 3:** Physical frailty, social frailty and oral frailty (total and factor scores): associations between frailty measures and dual task cost in animal naming and subtraction tasks.

	DTC Animal					DTC Subtraction				
	B (Unstd.)	SE	β (Std.)	p-value	R²	B (Unstd.)	SE	β (Std.)	p-value	R²
Physical Frailty (PF) – Fried Score										
PF Total	−0.443	0.983	−0.029	0.653	0.002	0.213	0.914	0.015	0.816	0.029
BMI <18.5	−2.321	4.710	−0.030	0.623	0.012	−3.257	4.379	−0.045	0.458	0.038
Fatigue	−4.305	2.602	−0.104	0.099		−2.663	2.419	−0.068	0.272	
Activity	0.922	1.843	0.032	0.617		1.514	1.713	0.056	0.378	
Gait speed	0.831	2.269	0.024	0.715		1.106	2.110	0.034	0.601	
Handgrip	0.281	2.700	0.006	0.917		.790	2.510	0.019	0.753	
Social Frailty (SF)										
SF Total	0.448	0.698	0.041	0.522	0.002	1.070	0.645	0.103	0.098	0.038
SF8 Factor 1 (Social resources)	−0.479	1.281	−0.024	0.709	0.005	−0.006	1.182	0.000	0.996	0.047
SF8 Factor 2 (Social activity and financial resources)	1.039	1.328	0.050	0.435		2.662	1.224	0.137	0.031*	
SF8 Factor 3 (Social need fulfilment)	1.084	1.813	0.037	0.550		0.277	1.672	0.010	0.869	
Oral Frailty (OF)										
OF Total	0.227	0.487	0.030	0.642	0.002	0.684	0.451	0.096	0.130	0.037
OFI-8 Factor 1 Swallowing difficulties	0.555	0.818	0.042	0.498	0.013	1.081	0.750	0.087	0.151	0.064
OFI-8 Factor 2 Dental Care	−0.881	0.878	−0.068	0.317		−0.905	0.806	−0.074	0.262	
OFI-8 Factor 3 Dietary and Social Habits	2.202	1.502	0.095	0.144		3.728	1.377	0.170	0.007*	

*p < 0.05.

Final composite model – Relationship between Frailty Measures versus CFAB with Dual Task Cost in Verbal Fluency and Arithmetic Tasks

The final composite model showed that CFAB remained the sole significant predictor of DTC for both arithmetic (B=−2.012, p < 0.001) and verbal fluency (B=−1.537, p = 0.016) tasks. (Table 4)

**Table 4:** Final composite model.

	DTC Animal					DTC Subtraction				
	B (Unstd.)	SE	β (Std.)	p-value	R²	B (Unstd.)	SE	β (Std.)	p-value	R²
Model: PF, SF, OF, CFAB										
PF	−0.828	0.990	−0.053	0.403	0.024	−0.310	.903	−0.021	0.731	0.082
SF	0.290	0.719	0.026	0.687		.749	.655	.072	0.254	
OF	0.090	0.502	0.012	0.857		.449	.458	.063	0.328	
CFAB	−1.536	0.649	−0.163	0.019		−2.011	.592	−0.226	<0.001*	

*p < 0.05.

Given that OFI-8 Factor 3 (Dietary and Social Habits) and SFS-8 Factor 2 (Social Activity and Financial Resources) share the item 'going out less frequently compared to the previous year,' multicollinearity was formally assessed in all models that included both instruments. Variance Inflation Factors (VIF) were calculated for each predictor; VIF values below 5.0 were considered acceptable [27]. All VIF values in the final composite model were within acceptable range (VIF range: 1.10–1.40), indicating that collinearity did not meaningfully inflate standard errors or destabilise the beta coefficients.

In addition, a sensitivity analysis was performed excluding the overlapping item (“going out less frequently compared to the previous year”) from the OFI-8. The modified OFI score yielded similar overall results, with overall results for the final composite model remains unchanged.

## 4 Discussion

This study highlights that executive dysfunction, rather than global cognition or physical frailty, is the primary determinant of DTC in cognitively intact older adults. By considering cognitive and multidimensional frailty domains together, the study reinforces the importance of executive function in dual-tasking. The associations between social and oral frailty with DTC, particularly in cognitively demanding tasks, also bring to light modifiable areas that may warrant future interventions.

### 4.1 Cognitive function and dual task performance

Consistent with previous literature on executive resource limitations [28,29], we found that executive dysfunction, as measured by CFAB is a key predictor of DTC across both verbal fluency and arithmetic tasks. Higher CFAB scores were associated with lower DTC in both tasks after adjusting for demographics and frailty dimensions. This shows that individuals with better executive function showed lower DTC, indicating greater resilience to cognitive-motor interference. This is likely due to more efficient resource allocation under dual task demands. This finding is consistent with the attentional resource theory of dual-task performance, which proposes that individuals with greater executive capacity are better able to allocate limited attentional resources between concurrent tasks, thereby maintaining gait stability under dual-task conditions [30]. Within this framework, individuals with preserved cognitive function may prioritise locomotor control more efficiently, resulting in lower dual-task cost. Supporting this, neuroimaging studies have shown that older adults with relatively intact prefrontal function exhibit more efficient and task-specific neural activation patterns during dual-task walking compared to those with executive impairment [31].

DTC for arithmetic tasks is more cognitively demanding and shows a positive association with both executive and memory function in our study. From a neurocognitive perspective, arithmetic tasks impose a higher level of cognitive demand compared to many standard dual-task paradigms. It requires continuous numerical manipulation, sequencing and monitoring for errors. These domains are tightly linked to executive function [32,33]. This greater cognitive load may explain why the arithmetic task more effectively revels subtle deficits in executive function that are not apparent under simpler conditions. In this context, our findings are consistent with the ‘Threshold of Vulnerability’ hypothesis, whereby dual-task interference becomes evident only when the task demands exceed an individual’s available cognitive resources [34].

Individuals with preserved executive function may be able to maintain gait performance by allocating sufficient attentional resources. However, those with reduced executive capacity, potentially related to early cognitive, social or oral frailty, are more likely to experience a decline in performance under higher task demands. This may account for the stronger associations observed with DTC arithmetic tasks compared to verbal fluency, which likely imposes a lower executive burden. This attenuation may be a result of the nature of semantic retrieval, which draws more heavily on semantic memory networks than on frontal-executive pathways [35]. The results suggest that dual task performance, especially under high cognitive load is sensitive to executive dysfunction and memory deficits.

Global cognitive status, as measured by CMMSE total score, was not significantly associated with DTC in either task. This shows that broad cognitive screening like the CMMSE [36] may lack sensitivity in detecting dual-task impairments. However, the CMMSE subdomain for recall did show a significant association with DTC arithmetic task indicating that specific cognitive functions such as memory recall may become vulnerable under greater cognitive load. This suggests that memory processes, especially delayed retrieval, may become vulnerable when cognitive load increases. This reinforces the concept that arithmetic dual tasks are more resource-intensive, requiring a broader and more demanding cognitive network.

Beyond identifying specific cognitive predictors of DTC, our findings highlight the clinical relevance of dual-task paradigms in the assessment of older adults. Unlike single-domain measures of cognition or gait, dual-task paradigms capture the interaction between motor and cognitive systems, which becomes increasingly disrupted with aging and frailty.

This disruption is thought to reflect a loss of automaticity in gait control. In healthy individuals, walking is largely automatic and requires minimal attentional input. With aging and emerging frailty, gait becomes more attentionally demanding, increasing reliance on executive control and competing with concurrent cognitive tasks for limited resources [37]. This competition, also known as the cognitive–motor interference, is more pronounced in populations with reduced processing speed and executive function [38].

By quantifying this interference, DTC provides a sensitive measure of functional vulnerability. It has been associated with adverse outcomes including falls [39], mobility decline [38], and cognitive deterioration [40], often beyond what can be detected using single-task assessments alone. Our findings emphasize that dual-task performance under high cognitive load is more sensitive to executive dysfunction and specific memory deficits, while tasks with low cognitive load may partially bypass these constraints by relying on more semantic retrieval processes [41].

This is also relevant to the concept of cognitive frailty (CF), defined as the coexistence of physical frailty and mild cognitive impairment in the absence of dementia. CF represents a state of increased vulnerability at the interface between cognitive and motor function, which is directly assessed by dual-task paradigms.

In this study, executive function (as measured by CFAB) was the strongest and most consistent predictor of DTC across both tasks, suggesting that cognitive impairment particularly in executive domains may play a central role in dual-task vulnerability than physical frailty alone in relatively high-functioning older adults.

Our findings are also relevant to the concept of cognitive frailty (CF), defined as the coexistence of physical frailty and mild cognitive impairment in the absence of dementia [3]. CF represents a state of increased vulnerability at the interface between cognitive and motor function, which is directly assessed by dual-task paradigms.

In this study, executive function (as measured by CFAB) was the strongest and most consistent predictor of DTC across both tasks, suggesting that cognitive impairment—particularly in executive domains—may play a more central role in dual-task vulnerability than physical frailty alone in relatively high-functioning older adults. In addition, the associations observed with social and oral frailty in the arithmetic task indicate that broader frailty domains may contribute to this vulnerability under higher cognitive load.

Taken together, these findings support a multidimensional view of cognitive frailty and suggest that dual-task cost may serve as a sensitive and functionally relevant marker for early cognitive-motor decline in community-dwelling older adults [42].

### 4.2 Frailty dimensions and dual task performance

Despite theoretic links to gait and physical capacity [18], no physical frailty component showed significance for both DTC verbal fluency or arithmetic tasks. Several factors may explain this finding. First, participants in the geriLABS-2 cohort were generally high-functioning, with most classified as robust or pre-frail. This limited severity and variability in physical frailty, which in turn reduced the ability to detect associations. Studies reporting significant relationships between physical and dual task performance often include individuals with more advanced frailty [43]. This may suggest that the cognitive load of the arithmetic tasks overshadows that gait-related burden. This is also a possible reflection of the relatively high baseline physical functioning in the sample population, indicating that physical frailty alone does not explain DTC once cognitive contributions are accounted for.

Second, the strong influence of executive function observed in our models may reflect the cognitive demands of the dual-task paradigms used [44]. Both serial subtraction and verbal fluency rely heavily on executive processes, which may overshadow the relatively modest impact of early physical decline on gait under dual-task conditions.

Lastly, physical frailty may represent a later-stage contributor to dual-task impairment that becomes more apparent only when motor control can no longer be maintained without increased executive support. This is a threshold that may not have been reached in this relatively robust cohort [45].

Social and oral frailty dimensions demonstrated selective associations with DTC in arithmetic tasks. Social Frailty Factor 2 (social activity and financial resources) was significantly associated with higher DTC in arithmetic tasks. Social engagement has been shown to support cognitive reserves through environmental stimulation and maintenance of cognitive function [46]. This aligns with theories that a socially integrated lifestyle supports cognitive reserve and buffers executive decline through enriched environmental stimulation [47,48]. Decreased life space, as reflected by going out less frequently compared to the previous year, and lack of commensality may underlie lower levels of social activity contributing to this vulnerability. Arithmetic tasks under dual-task conditions requires higher cognitive effort and social engagement (including financially support interactions) may help preserve executive functions like attention and working memory. Social inactivity or decreased financial resources could limit access to cognitively enriching experiences and psychosocial scaffolding, contributing to social isolation and subsequent decline in executive function [49].

Oral Frailty Factor 3 (dietary and social habits) was also significantly associated with higher DTC in arithmetic tasks. Reduced frequency of going out and problems with swallowing may relate to both social withdrawal and nutritional deficiencies, impacting cognition and metabolic energy needed for dual-tasking [50,51]. Poor dietary intake and masticatory difficulty have been linked to adverse brain changes and reduced cognitive abilities in older adults [52]. This aligns with earlier validation work by Nomura et al. [53], which found that check-list based oral frailty tools capture distinct dimensions, supporting the notion that non-dental oral factors reflect broader, frailty-related vulnerability.

These findings suggest that social vulnerability and nutritional or social isolation may amplify the cognitive burden of complex dual tasks potentially via mechanisms of reduced cognitive stimulation or unmet physiological needs. It is consistent with prior work and suggest that the oral-social frailty interface has a key functional impact on dual-task performance [54].

Such associations were not observed for the less demanding task of verbal fluency reinforcing the idea of task-specific susceptibility to frailty-related deficits.

### 4.3 Multidomain contribution to dual-tasking

The final composite model confirmed that CFAB total score remains the sole significant predictor of DTC across both verbal fluency and arithmetic tasks when adjusted for frailty dimensions. CFAB integrates both cognitive and behavioural control domains, and the findings suggest that executive function is the primary driver of dual-task performance in this cohort. This reinforces that executive function plays a central role in driving cognitive-motor interference. It supports prior work on dual-task paradigms in older adults [55,56].

However, the significance of specific social and oral frailty components in individual models for cognitively more demanding arithmetic tasks warrants continued attention. These domains may act as indirect modulators of executive function via mechanisms such as cognitive enrichment, psychosocial engagement or nutritional support. All of these are potentially modifiable through targeted interventions [51,57].

This supports a multidomain model in which executive function is the key determinant of dual-task ability, however this function is embedded within a broader matrix of frailty-related vulnerabilities. Addressing social and oral frailty, through community integration or meal programs, may enhance executive resilience and reduce dual-task deficits, in older adults who are at risk of cognitive decline.

These findings may also have several clinical implications on our comprehensive geriatric assessment (CGA). CGA has limitations in cognitive evaluation as it relies largely on global screening tools such as the CMMSE, which are designed to detect dementia and may not capture early executive dysfunction [58]. In addition, dual-task motor or cognitive assessments are not routinely included, indicating that integrated vulnerability reflected by DTC is not assessed in standard practice [59]. Tools like the CFAB may better capture early vulnerabilities in dual-tasking abilities that predict real world functional decline. It is feasible for use within a CGA setting [60]. Incorporating CFAB alongside dual-task assessment may provide a more comprehensive evaluation of cognitive motor vulnerability, enabling earlier identification of individuals at falls risk, functional decline and cognitive frailty [61].

Targeted assessment of social and oral frailty domains such as social participation, limitation of financial resources and feasibility of chewing hard food can highlight modifiable contributors to cognitive-motor interference. These areas are often under-assessed but can be feasibly integrated into the CGA to allow clinicians to identify older adults at higher risk of dual-tasking deficits. This supports a multidomain and proactive approach to geriatric care, with an emphasis on strengthening cognitive resilience and maintaining functional independence.

### 4.4 Limitations of the study and future work

The results of our study may not be generalizable to non-Asians or a frailer population. Cultural differences in education, nutrition and social engagement patterns may influence the relationship between frailty dimensions and dual-task performance.

Ethnicity was not included as a covariate in the present analyses due to the limited representation of non-Chinese participants. While ethnicity may influence both frailty and cognitive outcomes, the relatively homogeneous composition of the cohort is unlikely to have materially affected the observed associations.

The cross-sectional design precludes casual inference. While we observed associations between executive function, certain frailty subdomains and DTC, it is difficult to determine directionality or temporal sequencing.

The verbal fluency tasks also may not have imposed sufficient cognitive load to fully capture dual-task deficits in this group of participants who may be high-functioning. More demanding verbal tasks may enhance detection of DTC differences [62]. Tasks such as letter fluency (places greater demands on executive initiation) [63], alternating fluency tasks that require cognitive flexibility, and more complex serial subtraction (e.g. in steps of 3 or 7) [64]that increase working memory and monitoring demands. may provide greater sensitivity. The use of a graded set of cognitive tasks with varying levels of executive demand may allow more precise characterisation of the threshold at which dual-task interference emerges across different levels of frailty [65].

Our findings show the importance of executive dysfunction as the driver of dual-task impairments, with additional contributions from social and oral frailty domains during higher cognitive load conditions. This highlights the importance of multidomain assessments in identifying older adults at risk of functional decline. Future longitudinal studies should examine whether changes in social engagement, nutritional habits or cognitive training can influence DTC trajectories and whether targeting these domains can improve DTC and its related outcomes.

## 5 Conclusion

Our study demonstrates that executive dysfunction, as measured by CFAB, is the primary predictor of DTC in cognitively intact, community-dwelling older adults. In contrast, global cognition measured by CMMSE, was not significantly associated with DTC, suggesting that conventional screening tools may not capture the executive deficits underlying dual-task vulnerability.

These findings reflect the role of dual-task paradigms in assessing the cognitive–motor interface. As gait automaticity declines with aging and early frailty, locomotor control increasingly relies on executive resources, leading to competition with concurrent cognitive tasks. Individuals with reduced executive capacity are therefore more susceptible to cognitive–motor interference, particularly under higher task demands such as serial subtraction.

Physical frailty did not significantly predict DTC, likely reflecting the high functional baseline of participants or the dominant influence of cognitive load. Instead, our study highlighted the association of social (social activity and financial resources) and oral frailty (dietary and social habits) factors with greater DTC in cognitively demanding conditions, such as arithmetic tasks. This suggests that non-physical frailty domains may contribute to vulnerability through pathways related to reduced engagement and nutritional factors, indicating that executive dysfunction may represent a common pathway linking multidomain frailty to dual-task performance.

These findings have implications for clinical assessment. Incorporating executive function screening using CFAB, together with dual-task evaluation and assessment of social and oral frailty, may improve identification of older adults at risk of early cognitive–motor decline. Future longitudinal studies are needed to determine whether targeting these domains can improve dual task performance and delay functional decline.

## Funding

This research was funded by the Lee Foundation Grant 2019. The funder had no role in the design of the study; in the collection, analyses, or interpretation of data; in the preparation of the manuscript, or in the review or approval of the manuscript and in the decision to publish the results.

## Ethical standards

The National Healthcare Group Domain Specific Review Board.

(DSRB) granted ethics approval for this study (DSRB reference number: 2017/00850).

## Declaration of generative AI and AI-assisted technologies in the manuscript preparation process

During the preparation of this work, the authors used Chat Generative Pre-Trained Transformer (ChatGPT; OpenAI, San Francisco, CA, USA) for language editing for spelling and grammar check and to improve the logical flow and clarity of the manuscript. The AI tool was not used for data analysis or scientific conclusion. All content was reviewed and edited by the authors, who take full responsibility for the content of the publication.

## Data statement

Individual-level data from the GeriLABS-2 study cannot be shared due to institutional data governance restrictions. Aggregate data and summary statistics supporting the findings of this study are available from the corresponding author upon reasonable request.

## CRediT authorship contribution statement

**Louise Heyzer:** Writing – review & editing, Writing – original draft, Project administration, Methodology, Investigation, Formal analysis, Data curation. **Kristabella Low:** Writing – review & editing. **Cai Ning Tan:** Writing – review & editing. **Audrey Yeo:** Writing – review & editing. **Jia Qian Chia:** Writing – review & editing. **Justin Chew:** Writing – review & editing. **Joanne Kua:** Writing – review & editing. **Wee Shiong Lim:** Writing – review & editing, Supervision, Project administration, Methodology, Investigation, Funding acquisition, Data curation, Conceptualization.

## Declaration of competing interest

The authors declare no conflict of interest.
